# Toothmarks Made by a Burglar Lead to His Capture

**Published:** 1888-11

**Authors:** 


TOOTHMARKS MADE BY A BURGLAR LEAD TO HIS CAPTURE.—The circumstances of the detection of a colored man named Chandler Jones, who burglarized the store of J. L. Milton, of Hazlehurst, Ga., several weeks ago, are peculiar. The work was done by Detective E. A. Wilson, of Macon. It shows how that officer seized upon a slight clue and followed it to a successful result. When he arrived at Hazlehurst he made an examination of the store and found that the double door had been unlocked by the insertion of a chisel between the doors and gradually working the bolt into the lock. He then made an examination of the store, but saw nothing in the way of a clue except an apple out of which two bites had been taken. His detective instinct caused him to examine this apple, and he saw upon it toothmarks that were valuable. He saw that the two front teeth of the bites were not only irregular but peculiar. He imagined that when the biter was a boy an old tooth remaining in the gum caused a new tooth to grow one-sided, and it was now his resolve to find the man with that ingrowing tooth. The apple was placed in water so as to prevent shriveling, and keeping his secret to himself he went down to Baxley, where he knew there were a number of loafing negroes.

He found a group in a store and in the center of it was a real negro dude, and he was standing in an attitude that would have shamed a New York swell. The detective instinct came into play again and Mr. Wilson was certain that the dude was the man he wanted, but it was necessary to put him to the test.

Walking into the store, he bought a cigar. Then seeing some apples he bought a dime's worth, and, biting one, said to the crowd, "Here, I can't eat all these," and treated the group with the apples. His eyes were upon the dude, and when that individual took one bite the detective was dead sure of his man, and when he raised the apple to his mouth for a second bite the handcuffs were placed on his wrist. There never was a more astonished negro. He was under arrest so quickly that he was unable to offer any resistance, and submitted to the handcuffing. He was wearing a suit of clothes stolen from Mr. Bussey, of Chauncey, and a fine watch and chain taken from Mr. Milton. After placing Jones in the lock-up the detective found where he was stopping, and then he secured the valise with a number of Milton's goods, and in the valise were the little cards attached to the clothing, and on which were Milton's cost and selling marks.

Jones was then taken to Hazelhurst, and at the store showed how he went in with two others and made the haul. When he first went in on the night of the burglary the first thing he saw was a barrel of apples. He picked up one, and after two bites laid it down on Mr. Milton's desk. He owned up to the entire affair, and told where the other goods could be found. He also confessed to having robbed Mr. Bussey in Chauncey. And thus another apple caused man's downfall.

